# How the volatile organic compounds emitted by corpse plant change through flowering

**DOI:** 10.1038/s41598-022-27108-8

**Published:** 2023-01-07

**Authors:** Lili Kang, Jasmeen Kaur, Kelsey Winkeler, Daniella Kubiak, Jane E. Hill

**Affiliations:** 1 Thayer School of Engineering, Dartmouth College, Hanover, NH 03755 USA; 2 Department of Chemical and Biological Engineering, University of British Columbia, Vancouver, 2360 E Mall, Vancouver, BC V6T 1Z3 Canada

**Keywords:** Plant sciences, Gas chromatography

## Abstract

The corpse plant (*Amorphophallus titanum*) is so named because it produces a pungent, foul odor when flowering. Little is known about how the emitted volatiles change throughout the two-day flowering period. In this study, the comprehensive monitoring of the presence and change in volatile molecules during the female and the male flowering phases of *A. titanum* was conducted, and the plant temperature was monitored. A total of 422 volatile features were detected over the entire sampling period, of which 118 features were statistically significantly different between the pre-flowering and both flowering phases, and an additional 304 features were found present throughout the flowering period. A total of 45 molecules could be assigned putative names. The volatile profile of *A. titanum* changes over the two-day flowering period, with the S-containing molecules and aldehydes dominant in the female flowering phase, and the alcohols and hydrocarbons dominant in the male flowering phase. The two-dimensional gas chromatography time-of-flight mass spectrometry (GC × GC-TOFMS) enabled us to identify 32 new molecules produced by *A. titanum*. Each of these molecules alone, and in combination, likely contribute to the different odors emitted during the flowering phase of *A. titanum*.

## Introduction

When a plant is unable to fertilize itself effectively, it uses another method, such as the wind, insects, or birds to help it reproduce. A plant can attract insects and birds via the use of flower shapes, flower color pattern, and emission of odors^1^. One fascinating example of the effective use of odor attraction for the purpose of using insects to fertilize flowers is by the *Amorphophallus titanum*, a native of Western Sumatra and Western Java. *A. titanum* has the largest unbranched inflorescence in the world^2^, which makes it public favorite in botanical gardens around the world. The spadix of *A. titanum* extends up to 2.5 m in height and is dull yellow in color. The spathe is about 3 m in circumference, about 1.6 m in height, and is pale green in color with white spots on the outside and purplish-wine lines on the inside^3^. *A. titanum* is economically important for food and medicinal purposes. The underground storage stem (tuber) of *A. titanum* contains more glucomannan than the other Amorphophallus species, which helps in reduction of cholesterol levels, obesity and diabetes^3–5^.

When flowering, *A. titanum* emits a decay-like stench during its two-day flowering period^6^. The pulsing waves of pungent odors produced by a flowering *A. titanum* has led to it being referred to as the “corpse plant”^7^. The most common odors describe it as smelling like a rotting animal, a dead mouse, foul, and sulfur-like during flowering. Though produced simultaneously, the individual volatile molecules emitted during female flowering include: dimethyl disulfide (garlic-like odor^8^), dimethyl trisulfide (foul odor^9^), methyl thioacetate (sulfurous odor^10^), and isovaleric acid (cheesy, sweaty odor^10,11^). The diffusion of volatile molecules from the flowers is enhanced by thermogenesis. The spadix thermogenesis period starts after the opening of the spathe on the first day, reaching 36 °C, in pulses, synchronizing with the waves of the carrion-like odor^12^. The thermogenesis of male flowers begins on the second day when pollens are being released, where the temperature of the florets can also reach up to 36 °C^13^. The flowering *A. titanum* draws insects that are typically attracted to carrion, including dung beetles and flesh flies^9^.

Little is known about the variety and abundance of the volatile molecules and odors emitted by *A. titanum* over time and publications appear to only focus on the female flowering phase^8,10^. In the work presented here, we undertake a comprehensive survey of the molecules produced by *A. titanum* before and during the female and the male flowering phases, an effort complimented by thermogenesis measurements.

## Methods

### Sampling of volatile molecules

Volatile molecules were sampled from an *A. titanum* grown in Dartmouth College’s Life Sciences Greenhouse during early November. Two samples were collected per day before the plant started flowering. When the spathe started to open and the odor became noticeable on the third day, samples were taken approximately every 2 h at a spot between the spadix and the top of the spathe. Each sample was drawn onto a thermal desorption tube (150 mL/min for 10 min; Carbopack Y, X, and Carboxen 1000 [Supelco, Bellefonte, PA])^14^. The thermal desorption tubes were hermetically sealed immediately after collection and stored at 4 °C for up to two weeks until analysis. A total of 16 samples were taken during this project.

### Thermal imaging

Thermal images were taken at each volatile molecule sampling time point using a FLIR E60 thermal camera (FLIR, Wilsonville, OR) with a focal length of 18 mm and an exposure time of 1/59 s. For each image, the camera was placed at the same two locations. At each location, two angles of elevation were used, leading to four thermal images being taken at each time point.

### Analytical instrumentation

The volatile molecules concentrated on the thermal desorption tubes were analyzed by a Pegasus 4D (LECO Corporation, St. Joseph, MI) GC × GC-TOFMS instrument with an Agilent 6890 GC equipped with a Gerstel Thermal Desorption Unit (TDU), a Gerstel CIS 4 cooled injection system, and a Multi-Purpose Sampler (Gerstel, Linthicum Heights, MD). The TDU desorption temperature was programmed to increase from 50 °C (held for 0.5 min) to 300 °C (held for 5 min) at 7 °C/min, and desorbed molecules were cryo-focused at −30 °C in the CIS inlet. Analytes were then introduced into the capillary column by ramping the CIS inlet temperature from −30 °C (held for 3 min) to 330 °C (held for 3 min) at 12 °C/s in splitless mode.

Chromatographic analysis was performed using a Rxi-624Sil (60 m × 250 μm × 1.4 μm) as the first dimension (1D)-GC column and a Stabilwax (1 m × 250 μm × 0.5 μm) as the second dimension (2D)-GC column, both purchased from Restek (Bellefonte, PA). High purity helium (Airgas, Radnor, PA) was used as the carrier gas with a flow rate at 3 mL/min. The primary oven was held at 40 °C for 2 min, and then increased to 235 °C (held for 3 min) at a rate of 3 °C/min. The temperature offsets were + 5 °C for the secondary oven and + 20 °C for the modulator. The modulation time was 2.5 s total, alternating 0.85 s hot and 0.40 s cold pulses. TOFMS was employed as a detector, with electron ionization at 70 eV, ion source maintained at 200 °C, and mass range of 40–400 m/z monitored at an acquisition rate of 150 spectra/s. Data acquisition and analysis were performed using ChromaTOF software, version 4.70.7.0 (LECO Corporation).

### Processing and analysis of chromatographic data

Chromatographic data were processed and aligned using ChromaTOF. For peak finding, a signal-to-noise cutoff (S/N) was set at 100:1 (a minimum of three apexing masses) in at least one chromatogram and a minimum of 50:1 S/N in all others. The resulting peaks were identified by a forward search of the NIST 2011 library. For putative peak identification, a forward match score of ≥ 850 (of 1000) was required. For the alignment of peaks across the chromatograms, the maximum 1D and 2D retention time deviations were set at 7.5 s and 0.15 s, respectively, and the inter-chromatogram spectral match threshold was set at 600. The molecules eluting prior to 200 s in the first dimension and 0.4 s in the second dimension were removed prior to statistical analysis using the Classification feature in ChromaTOF^15^. A data cleaning step was performed to remove suspected chromatographic artifacts and common environmental contaminants, as defined previously^16^.

### Statistical analyses

All statistical analyses were performed using R v3.5.3 (R Foundation for Statistical Computing, Vienna, Austria). Prior to statistical analyses, the relative abundance of molecules across chromatograms was normalized using probabilistic quotient normalization (PQN)^17^ and peak intensities were log-transformed, mean-centered, and then unit-scaled. The Mann–Whitney U test^18^ was used to select volatile molecules that were statistically significantly different between the flowering phases, with a p-value of 0.05 as the threshold for statistical significance.

### Ethics statement

We declare that the work reported here is consistent with the IUCN Policy Statement on Research Involving Species at Risk of Extinction.

## Results and discussion

### Observation of temperature change during flowering

**Figure 1:**
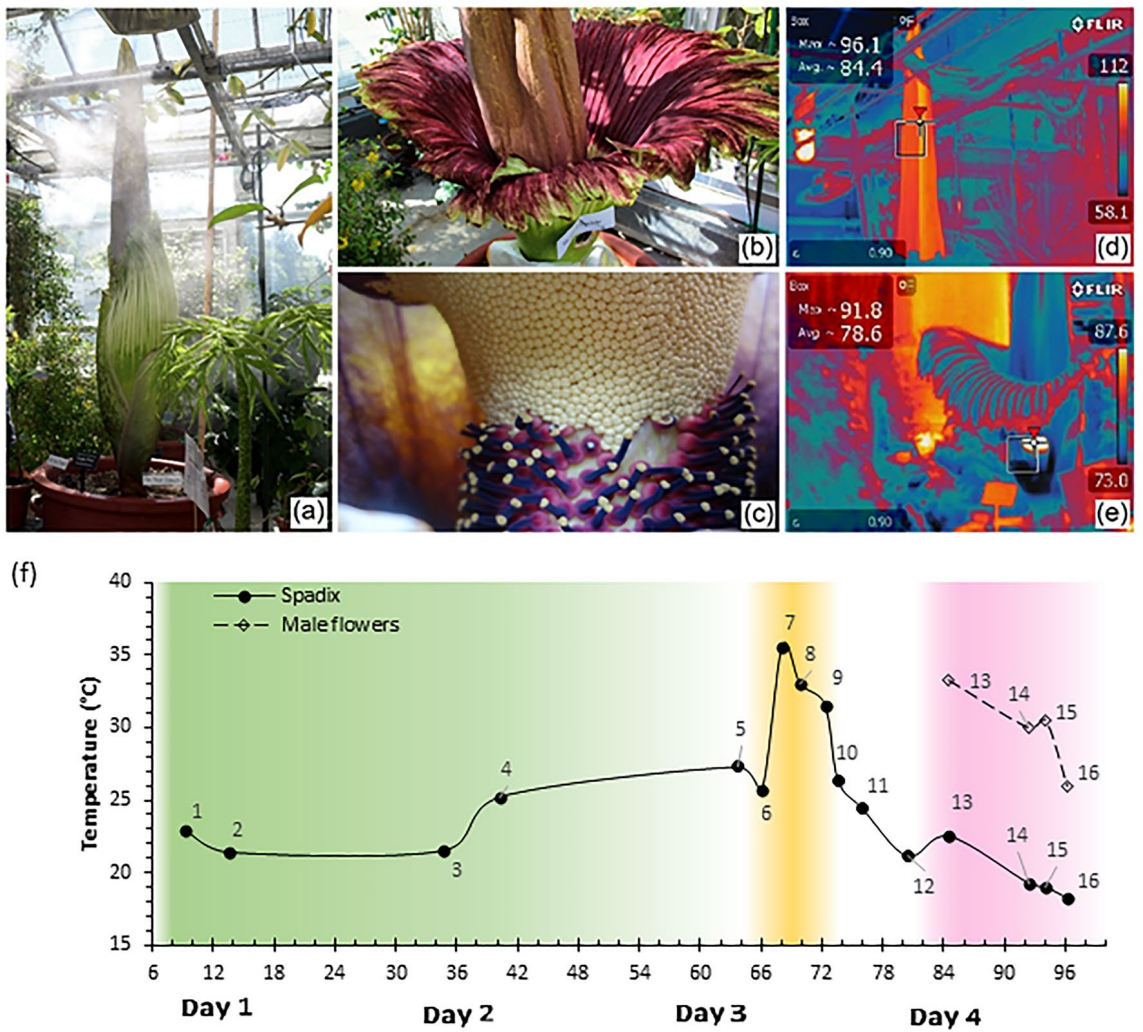
Temperature and odor changes during *A. titanum* flowering: Image of *A. titanum* (**a**) before flowering, (**b**) during flowering, and (**c**) male and female flowers; (**d**) thermal image of the spadix during the female flowering phase; (**e**) thermal image of the florets during the male flowering phase; (**f**) temperature profile of the spadix and the male flowers. The numbers 1–16 represent the samples collected at different time points.

The *A. titanum* (Fig. 1a–c) was sampled for temperature and volatile molecules over four days. Thermal profiling was used to help determine the onset and end of the female and male flowering periods^12,13^ (Fig. 1d, e). The highest female and male flowering temperatures were 35.6 °C and 33.2 °C, respectively. The maximum measured temperature during male flowering was 2.8 °C lower than expected^13^, either due to our 2 h sampling window being too coarse, causing us to miss the maxima, or due to a small window cut by the greenhouse staff to conduct hand pollination. The designation of the onset and end of the female and male flowering is projected onto Fig. 1f. The volatile molecules sampling was staged into three categories: pre-flowering, female flowering, and male flowering. All other samples were considered transition and not included in statistical analyses.

### Volatile profiles of *A. titanum* during the pre-flowering and the flowering phases

A total of 422 volatile features were detected in all 16 samples collected during the pre-flowering and the flowering phases of *A. titanum*. Of these, 118 features were statistically significantly different between the pre-flowering and the flowering phases (*p* ≤ 0.05), of which 26 features could be assigned putative names (Table 1).

**Table 1:** Putatively identified volatile molecules that are statistically significantly different between the pre-flowering and the flowering phases.

Volatile molecule	Formula	Class	Phase comparison^a^ (dominant phase↑)	RI mean^b^	Relative abundance^c^
3-Methylbutanal	C_5_H_10_O	Aldehyde	PvF (F↑)	705	0.043
Limonene	C_10_H_16_	Hydrocarbon	PvF (P↑)	1047	0.034
Methanethiol	CH_4_S	S-containing	PvF (F↑); FvM (F↑)	579	0.115
Methyl thiolacetate	C_3_H_6_OS	S-containing	PvF (F↑); FvM (F↑)	735	0.219
Dimethyl disulfide	C_2_H_6_S_2_	S-containing	PvF (F↑); FvM (F↑)	779	2.622
Dimethyl trisulfide	C_2_H_6_S_3_	S-containing	PvF (F↑); FvM (F↑)	1011	0.507
Dimethyl sulfide	C_2_H_6_S	S-containing	FvM (F↑)	616	0.175
2-Propenenitrile	C_3_H_3_N	N-containing	FvM (M↑)	627	0.028
2-Methylhexane	C_7_H_16_	Hydrocarbon	FvM (M↑)	684	0.120
2-Methyl-1-propanol	C_4_H_10_O	Alcohol	FvM (M↑)	691	4.709
Amylene hydrate	C_5_H_12_O	Alcohol	FvM (M↑)	696	1.329
1-Butanol	C_4_H_10_O	Alcohol	FvM (M↑)	720	0.023
2-Methyl-1-propyl formate	C_5_H_10_O_2_	Ester	FvM (M↑)	724	0.067
2-Pentanone	C_5_H_10_O_2_	Ketone	FvM (M↑)	736	0.129
2,4-Dimethylhexane	C_8_H_18_	Hydrocarbon	FvM (M↑)	740	0.652
Tetrahydro-2,2,5,5-tetramethylfuran	C_8_H_16_O	Ether	FvM (M↑)	781	0.089
2,4-Dimethyl-1-heptene	C_9_H_18_	Hydrocarbon	FvM (M↑)	848	1.281
Benzaldehyde	C_7_H_6_O	Aldehyde	FvM (F↑)	1022	0.059
Nonanal	C_9_H_18_O	Aldehyde	FvM (F↑)	1148	0.184
2-Butanol	C_4_H_10_O	Alcohol	FvM (M↑); MvP (M↑)	666	0.034
3-Methylhexane	C_7_H_16_	Hydrocarbon	FvM (M↑); MvP (M↑)	692	0.245
2-Methyl-2-(1-methylethoxy)propane	C_7_H_16_O	Ether	FvM (M↑); MvP (M↑)	721	0.054
3-Methyl-3-buten-1-ol	C_5_H_10_O	Alcohol	FvM (M↑); MvP (M↑)	783	0.272
2,4-Dimethylheptane	C_9_H_20_	Hydrocarbon	FvM (M↑); MvP (M↑)	824	0.489
2-Methyl-1-pentene	C_6_H_12_	Hydrocarbon	MvP (M↑)	635	2.799
3-Methyl-1-butanol	C_5_H_12_O	Alcohol	MvP (M↑)	787	0.044

Data from 16 samples. Only peaks that were above the pre-defined detection criteria were included in the statistical calculations.

^a^Statistically different volatiles in the pre-flowering versus the female flowering (PvF), the female versus the male (FvM), and the male versus the pre-flowering phases (MvP). The *p*-values were not corrected for multiple comparison adjustments.

^b^Linear retention index was determined using C_8_–C_20_ n‐alkane standard solution on a Rxi-624Sil (60 m × 250 μm × 1.4 μm) column. RI below 800 was extrapolated by ChromaTOF.

^c^Relative abundance = analyte total peak area / total peak area of all analytes detected.

### Dominant volatile molecules exuded during the female flowering phase

**Figure 2:**
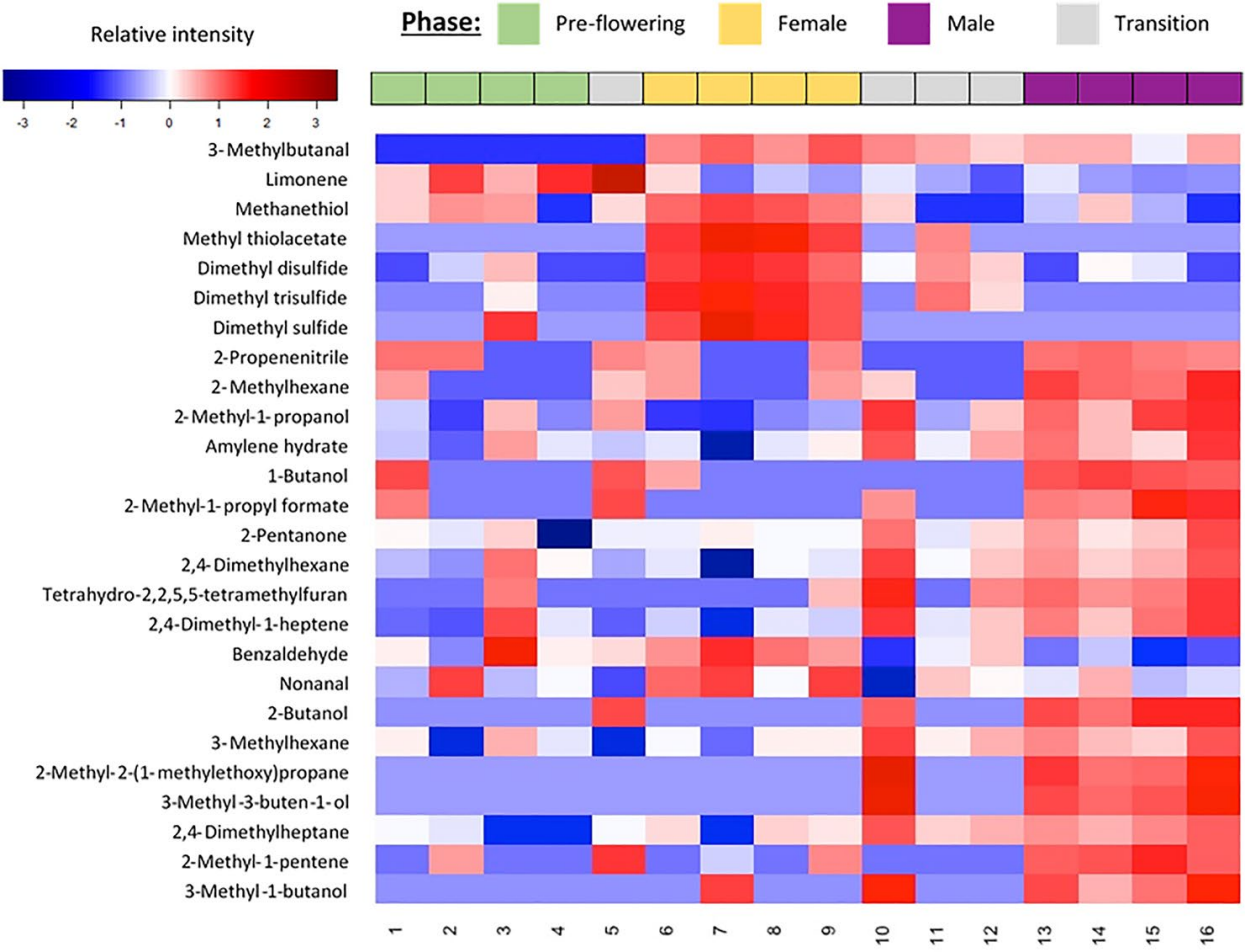
Heat map of the inflorescence’s scent profile over the pre-flowering and the flowering phases using statistically significant molecules.

Eight volatile features were statistically significantly different between the pre-flowering and the female flowering samples (*p* ≤ 0.05). Putative names could be assigned to six features (Table 1), of which one feature, identified as limonene, has been detected for the first time in *A. titanum*. The change in abundance of these six molecules over the entire sampling period is visualized using a heat map (Fig. 2), which shows five of the six being more abundant during female flowering, with sulfur-containing molecules dominating.

During the female flowering phase, dimethyl disulfide (DMDS) was present in the highest abundance. DMDS was also detected, albeit at a lower intensity and infrequently, in the pre-flowering and the male flowering phase (Fig. 2). DMDS has been previously reported as a dominant chemical component of *A. titanum* scent, with an odor generally described as garlic-like^8–10,19^. Dimethyl trisulfide (DMTS) was present in the second highest relative abundance, with peak emission during the female flowering phase and decreasing during the female to male transition. DMTS was not detected during the male flowering phase (Fig. 2). DMTS’s decayed cabbage, decayed meat and garlic smell is considered a main odor contributor for *A. titanum*^8,10,19^ with a very low odor threshold^20^.

Methyl thiolacetate was the third most abundant molecule, followed by methanethiol and 3-methylbutanal. The level of these three molecules was highest during the female flowering phase and was lower or undetected in the other phases (Fig. 2). Methyl thiolacetate has a cheesy, garlic smell^21^ and has been detected in the floral scent of *A. titanum*^10^. Methyl thiolacetate in combination with DMTS was attractive to adult carrion beetles (*Necrophila americana* and *Oiceoptoma noveboracense*)^22^. Methanethiol (sulfurous odor^23^) was released less than the other S-containing molecules (Table 1). Methanethiol is the precursor for biosynthesizing DMDS in plants^24^. 3-Methylbutanal was present in high abundance during the female flowering phase, and remained somewhat abundant thereafter (Fig. 2). 3-Methylbutanal (malty odor^23^) has been detected in the headspace of the fluid secreted from the spadix of *A. titanum* during female flowering^10^. Limonene was most abundant during the pre-flowering period, reaching its maximum just prior to the start of the female flowering phase and then dropping to barely detectable levels thereafter (Fig. 2). Although the citrus-scent of limonene has been detected in the floral scent of other Amorphophallus species, such as *A. arnautovii*, *A. maximus* and *A. Zenkeri*^8,9^, it is possible that the limonene monitored here came from a geranium flowering nearby at the time^25^.

One hundred features were statistically significantly different between the female and the male flowering phase samples (*p* ≤ 0.05). Of these, five features were overlapping with those found statistically significantly different between the pre-flowering and the female flowering samples, and 95 features were found new in this comparison. Of the 100 features, putative names could be assigned to 22 molecules (Table 1), among which seven (S-containing and aldehydes) molecules were found dominant in the female flowering phase (Fig. 2). Among the S-containing molecules, DMDS, DMTS, methyl thiolacetate and methanethiol, which were dominant in the female flowering phase in comparison to the pre-flowering phase, remained statistically significant and dominant in the female flowering phase in comparison to the male flowering phase, with the presence of dimethyl sulfide (cabbage-like odor^23^) being a new addition to this family. DMDS and methanethiol were present in lower abundances during the male flowering period but still likely contributing to the rotting/dead animals smell during that period as they have a low odor threshold. Aldehyde emission patterns were similar to the S-containing molecules, and had higher abundances in the female flowering phase. Nonanal (citrus and fatty odor^26^) and benzaldehyde (almond odor^26^) have been found previously in the appendix of *A. titanum*^21^. Benzaldehyde has been found attractive to *Diabrotica virgifera* beetle^27^.

### Dominant volatile molecules exuded during the male flowering phase

Among the 22 putatively identified molecules, which were statistically significantly different between the female and the male flowering samples, 15 molecules were found dominant in the male flowering phase. Their abundance is visualized in Fig. 2, with alcohols and hydrocarbons dominating.

Within the alcohol group, 2-methyl-1-propanol, amylene hydrate, 2-butanol, 1-butanol and 3-methyl-3-buten-1-ol were the new volatiles identified in *A. titanum* during this study. 2-Methyl-1-propanol (isobutanol), which smells like sweet alcohol^28^, was present at the highest relative abundance, followed by amylene hydrate (tert-amyl alcohol; camphoraceous odor^29^), 3-methyl-3-buten-1-ol (isoprenol; fruity odor), 2-butanol (fruity odor) and 1-butanol (fruity odor). Various species of social wasps in the *Vespula* and *Dolichovespula* genera (yellow jackets) are attracted to 2-methyl-1-propanol, and the attraction extends to paper wasps (*Polistes fuscatus*) when 2-methyl-1-propanol is combined with acetic acid^30^. 3-Methyl-3-buten-1-ol (isoprenol) is a known metabolite of isopentenyl pyrophosphate, an isoprenoid precursor in the biosynthesis of terpenes and terpenoids in plants^31,32^. 2-Butanol and 1-butanol have been reported as the main constituents in the odor profile of *A. pilosus* and *A. henryi*, respectively^20,33^. 2-Butanol is a known aggregation pheromone of the male rhinoceros beetle, a coconut tree pest^34^. It has also been found as one of the attractants for two beetles, *Parastasia bimaculata* and *Chaloenus schawalleri* (Chrysomelidae)^35^. *P. bimaculata* is known to be attracted to heat-generating, odor-producing flowers, whereas *C. schawalleri* is less selective about the heat-generation. 1-Butanol is among the most abundant molecules in *Petunia integrifolia* and *P. secreta* pollens that attracts short-tongued bees of family Halictidae^36^.

The hydrocarbons are the second largest group among the named molecules. The long-chain n-alkanes (C13, C14, C25 to C28) have been reported as being produced by the female flowers of *A. titanum* previously^37^. This is, however, the first time the volatile presence of the branched chain hydrocarbons (Table 1) is being reported for *A. titanum*, which includes 2-methylhexane, 2,4-dimethylhexane, 2,4-dimethyl-1-heptene, 3-methylhexane, 2,4-dimethylheptane and 2-methyl-1-pentene. Some of these branched hydrocarbons have, however, been detected as volatiles produced by other plants^38–41^. 2-Methylhexane has been found in the volatile profile of a rose variety, Red Eagle^40^. 2,4-Dimethylhexane and 2,4-dimethyl-1-heptene have been identified in the volatile profiles of *Gentiana asclepiadea* leaves^39^ and flesh of *Citrus grandis* fruit^38^. 3-Methylhexane was also found, in lesser amount, in the pre-flowering phase. 3-Methylhexane is reported to be induced as a defense volatile molecule from an infested cotton plant (*Gossypium hirsutum*) against a green lacewing predator, *Chrysoperla lucasina*^40^.

Among ethers, ketones, esters and N-containing molecules, tetrahydro-2,2,5,5-tetramethylfuran, 2-methyl-2-(1-methylethoxy)propane, 2-methyl-1-propyl formate, 2-propenenitrile and 2-pentanone were the new molecules identified in *A. titanum*. Tetrahydro-2,2,5,5-tetramethylfuran is a known volatile molecule produced by the fungi *Cladosporium cladosporioides* that promotes plant growth^42^. 2-Propenenitrile has been detected as released by enophytic fungi, showing antifungal activity against *Monilinia fructicola*, a fungal plant pathogen^43^. It is possible that these two molecules monitored here were produced by microorganisms or perhaps produced by the plant itself. 2-Pentanone is the only ketone identified in the male flowering phase, and has been reported in the inflorescence odors of other Amorphophallus species, such as *A. borneensis*, *A. eichleri* and *A. obscurus*^44^.

The statistical analysis between the male flowering and the pre-flowering samples showed that 74 features were statistically significantly different (*p* ≤ 0.05). Of these, two features were overlapping with those found statistically significantly different between the pre-flowering and the female flowering samples, 57 features were overlapping with the statistically significantly different features between the female flowering and the male flowering samples, and 15 features were found new in this comparison. Of the 74 features, putative names could be assigned to seven molecules (Table 1) which were present at higher amounts in the male flowering phase, with alcohols and hydrocarbons dominating. Among these molecules, 2-butanol, 3-methylhexane, 2-methyl-2-(1-methylethoxy)propane, 3-methyl-3-buten-1-ol and 2,4-dimethylheptane were also found dominant in the male flowering phase during the comparison with the female flowering phase. 2-Methyl-1-pentene and 3-methyl-1-butanol were the additional molecules found dominant in the male flowering phase, and have been detected for the first time in the volatile profile of *A. titanum*. 2-Methyl-1-pentene was present at the highest relative abundance among all the hydrocarbons. 3-Methyl-1-butanol (isoamyl alcohol; fruity odor) has been reported previously in the inflorescence odors of *A. borneensis*, *A. commutatus* and *A. konjac*, as well as *Aristolochia microstoma* flowers^44,45^.

Some studies have shown that the inflorescence of a plant produces different smells when injured^46^. In this study, before sample 8 was collected, a small window was cut into the bottom of the spathe of *A. titanum* for the greenhouse staff to conduct hand pollinations (Fig. 1e). After the window was made, production of the S-containing molecules and aldehydes dropped sharply, though they increased subsequently. The intensity of most of the alcohols and hydrocarbons increased instead, before being reduced to much lower levels, and rising again during the male flowering phase. It was not clear whether the injury was the cause for the sudden change of volatiles in sample 10.

### Other volatile features of interest

In addition, 304 other features were present throughout the flowering period of *A. titanum*, with hydrocarbons, aldehydes and ketones dominating. These statistically non-significant molecules, also likely contribute to the odors emitted during the pre-flowering and the flowering phases. Putative names could be assigned to 19 molecules in this group as shown in Table 2, of which 14 molecules were found for the first time in *A. titanum* during this study. These include, benzene, octane, 3-tridecene, butyl benzene, pentyl benzene, hexyl benzene, hexanal, octanal, pentanal, heptanal, acetone, 2,3-butanedione, 2-butanone and tetrahydrofuran.

**Table 2:** Putatively identified volatile molecules that are statistically non-significant between the pre-flowering and the flowering phases.

Volatile molecule	Formula	Class	RI Mean^a^	Relative abundance^b^
Toluene	C_7_H_8_	Hydrocarbon	794	0.229
Benzene	C_6_H_6_	Hydrocarbon	691	0.181
Heptane	C_7_H_16_	Hydrocarbon	714	0.065
Octane	C_8_H_18_	Hydrocarbon	803	0.027
3-Tridecene	C_13_H_26_	Hydrocarbon	1497	0.013
Butyl benzene	C_10_H_14_	Hydrocarbon	1084	0.011
Pentyl benzene	C_11_H_16_	Hydrocarbon	1186	0.011
Hexyl benzene	C_12_H_18_	Hydrocarbon	1289	0.005
2-Propenal	C_3_H_4_O	Aldehyde	602	0.113
Hexanal	C_6_H_12_O	Aldehyde	840	0.050
Octanal	C_8_H_16_O	Aldehyde	1045	0.038
Pentanal	C_5_H_10_O	Aldehyde	740	0.024
Butanal	C_4_H_8_O	Aldehyde	656	0.019
Heptanal	C_7_H_14_O	Aldehyde	942	0.017
Acetone	C_3_H_6_O	Ketone	591	3.471
2,3-Butanedione	C_4_H_6_O_2_	Ketone	654	0.121
2-Butanone	C_4_H_8_O	Ketone	659	0.146
Ethanol	C_2_H_6_O	Alcohol	587	0.023
Tetrahydrofuran	C_4_H_8_O	Ether	674	0.009

The *p*-values were not corrected for multiple comparison adjustments.

^a^Linear retention index was determined using C_8_–C_20_ n‐alkane standard solution. RI below 800 was extrapolated by ChromaTOF.

^b^Relative abundance = analyte total peak area/total peak area of all analytes detected.

The hydrocarbons mainly consisted of n-alkanes and alkylated benzenes (cyclic hydrocarbons), with toluene present at the highest abundance (Table 2). Toluene and heptane have been previously found in the scent of *A. titanum* inflorescence, with glue solvent-like and gasoline-like smell, respectively^9^. Toluene was also detected in the pollen aroma of *Petunia integrifolia* and *Petunia secreta*^36^. Benzene has been detected, in a low amount, in the floral scent of *Gelsemium sempervirens*^47^. Octane has been found among the aroma components of sweet cherry (*Prunus avium* L.) flower essential oils^48^. 3-Tridecene and pentylbenzene have been reported in the essential oil of *Haplophyllum tuberculatum* leaves and stem^49^. Butylbenzene has been detected in the volatile profiles of *Gentiana asclepiadea* flowers^39^. Pentylbenzene is reported as a component in the essential oil of aerial parts of *Kellusia odoratissima* (wild celery) ecotypes^50^.

Among the aldehydes, 2-propenal (acrolein) was present at the highest abundance (Table 2). It has been found as one of the indicators of *A. titanum* inflorescence blooming^51^. Hexanal (citrus, orange odor^52^) has been reported as a minor volatile molecule in *A. pilosus*^33,44^. Octanal, along with heptane, 3-methylhexane, nonanal, octanal and other volatiles, has been identified as a semiochemical produced in *Gossypium hirsutum* for protection against predatory *Chrysoperla* species^41^. Octanal has also been found in the essential oil of *H. tuberculatum* aerial parts, along with 3-tridecene and pentylbenzene^49^. Pentanal (valeraldehyde) has been found in ‘Fuego’ variety of rose^40^, along with hexanal, heptanal and octanal among other volatile molecules. Butanal (butyraldehyde; rancid, sweaty odor) has been reported in the floral odors from *A. titanum*^23^. Heptanal (citrus, orange odor^52^) is released in caterpillar-infested plant, *Arabidopsis thaliana*, along with 1-butanol, 2-pentanone, nonanal, heptanal and other volatiles, to attract *Cotesia rubecula* parasitoid in the defense mechanism against *Pieris rapae* caterpillars^53^.

Acetone was found at the highest abundance among all the statistically non-significant molecules (Table 2). It has been reported in various other Amorphophallus species, such as *A. borneensis*, *A. commutatus*, *A. eburneus*, *A. erythrrorhachis*, *A. henryi*, *A. konjac*, *A. macrorhizus*, *A. plicatus* and *A. tinekeae*^33,44^. 2-Butanone has been identified as a scent molecule in *A. konjac*^33^. 2,3-Butanedione (butter-like odor), along with heptanal and hexanal, has been found among the contributors to *Symplocarpus renifolius* floral scent during the female, bisexual, and male flowering phases^52^. Ethanol was the only alcohol identified throughout the flowering phase of *A. titanum* in the present study. It was previously found in the scent profile of other Amorphophallus species, such as *A. cirrifer*, *A. konjac* and *A. obscurus*^33,44^. Ethanol has also been detected among the major volatiles present in the pollen aroma of *Petunia integrifolia* and *Petunia secreta*^36^.

### Study limitations

Samples in this study were from only one flowering *A. titanum* which was located in a greenhouse filled with other plants, a small number of which were concurrently flowering. In this case, isolating the plant from others was not possible, however, once the plant started flowering, we are reasonably confident that the sampling was dominated by *A. titanum* sources based on the proximity of the sampler and the natural convection from thermogenesis. We note also that the greenhouse was illuminated by a warm, strong (100 W) light bulb each night, which may have impacted the plant’s behavior. Importantly, while we reached level 2 of the naming convention outlined by the Metabolomics Standards Initiative (MSI)^54,55^, the molecules listed herein are only putative identities, which need to be verified using authentic standards.

## Conclusion

The present study represents the first time series analysis of the volatile molecules emitted by *A. titanum* using GC × GC-TOFMS. By analyzing 16 samples collected over the flowering period, 422 volatile features were detected. Of these, 118 features were statistically significantly different between the pre-flowering phase and both flowering phases. A total of 26 of these molecules were assigned with putative identifications based on their mass spectral matching, of which 18 molecules are first time ever reported in *A. titanum*. The female flowering phase was dominated by the S-containing molecules and aldehydes, and the alcohols and hydrocarbons were dominant during the male flowering phase. While the S-containing molecules are the major contributors to the foul odor emitted during the female flowering phase, the aldehydes, alcohols, hydrocarbons and ketones also contribute to the unpleasant odor of the corpse flower. Some of these volatile molecules have been reported to play an important role in plant–insect interactions, and could be responsible for attracting the pollinators and inducing the defense mechanisms in *A. titanum*. The additional 304 features were found present during the entire flowering period, and 19 of these molecules were assigned putative names which also likely contribute to the putrid odor of the corpse flower. Of these 19 molecules, 14 molecules are the newly identified in *A. titanum* in this study. This study demonstrated that *A. titanum* not only emitted odorants in the female flowering phase, but also a large number and wide variety of volatile molecules in the male flowering phase.

## Data Availability

The datasets generated and/or analyzed during the current study are available on reasonable request to the corresponding author.
